# Non-Randomized Trial of Dornase Alfa for Acute Respiratory Distress Syndrome Secondary to Covid-19

**DOI:** 10.3389/fimmu.2021.714833

**Published:** 2021-10-20

**Authors:** Zachary M. Holliday, Alexander P. Earhart, Mohammed M. Alnijoumi, Armin Krvavac, Lee-Ann H. Allen, Adam G. Schrum

**Affiliations:** ^1^ Department of Medicine, University of Missouri, Columbia, MO, United States; ^2^ Molecular Pathogenesis and Therapeutics Program, University of Missouri, Columbia, MO, United States; ^3^ Department of Molecular Microbiology and Immunology, University of Missouri, Columbia, MO, United States; ^4^ Department of Surgery, University of Missouri, Columbia, MO, United States; ^5^ School of Medicine, Biomedical Biological, and Chemical Engineering, University of Missouri, Columbia, MO, United States

**Keywords:** pneumonia, viral, respiratory failure, ARDS, COVID - 19

## Abstract

**Background:**

The most severe cases of Coronavirus-Disease-2019 (COVID-19) develop into Acute Respiratory Distress Syndrome (ARDS). It has been proposed that oxygenation may be inhibited by extracellular deoxyribonucleic acid (DNA) in the form of neutrophil extracellular traps (NETs). Dornase alfa (Pulmozyme, Genentech) is recombinant human deoxyribonuclease I that acts as a mucolytic by cleaving and degrading extracellular DNA. We performed a pilot study to evaluate the effects of dornase alfa in patients with ARDS secondary to COVID-19.

**Methods:**

We performed a pilot, non-randomized, case-controlled clinical trial of inhaled dornase for patients who developed ARDS secondary to COVID-19 pneumonia.

**Results:**

Improvement in arterial oxygen saturation to inhaled fraction of oxygen ratio (PaO_2_/FiO_2)_ was noted in the treatment group compared to control at day 2 (95% CI, 2.96 to 95.66, P-value = 0.038), as well as in static lung compliance at days 3 through 5 (95% CI, 4.8 to 19.1 mL/cmH_2_O, 2.7 to 16.5 mL/cmH_2_O, and 5.3 to 19.2 mL/cmH_2_O, respectively). These effects were not sustained at 14 days. A reduction in bronchoalveolar lavage fluid (BALF) myeloperoxidase-DNA (DNA : MPO) complexes (95% CI, -14.7 to -1.32, P-value = 0.01) was observed after therapy with dornase alfa.

**Conclusion:**

Treatment with dornase alfa was associated with improved oxygenation and decreased DNA : MPO complexes in BALF. The positive effects, however, were limited to the time of drug delivery. These data suggest that degradation of extracellular DNA associated with NETs or other structures by inhaled dornase alfa can be beneficial. We propose a more extensive clinical trial is warranted.

**Clinical Trial Registration:**

ClinicalTrials.gov, Identifier: NCT04402970.

## Introduction

Health care systems across the world are being inundated with patients who are critically ill due to infection with Severe Acute Respiratory Syndrome-Coronavirus-2 (SARS-CoV-2) causing Coronavirus Disease 2019 (COVID-19). Around 10% of those infected will develop the most severe manifestation of the disease requiring admission to an intensive care unit (1). Early reports suggested neutrophil extracellular traps (NETs) as being a potential contributor to the severity of disease in some patients with COVID-19 (2). As originally described, NETs were proposed to ensnare and potentially kill invading microbes, but it is now clear that these extracellular DNA-protein complexes are almost always deleterious and contribute to tissue destruction and pathogenesis in many diseases (3, 4). In severe cases of COVID-19, NETs appear to cause significant morbidity in the lungs with associated microthrombi formation, endothelial damage, capillaritis, neutrophilic mucositis and mucus accumulation (2, 5–7). Plasma levels of NETs are increased in patients requiring intubation and are inversely correlated with arterial blood oxygen content to fraction of inspired oxygen ratio (PaO_2_/FiO_2_) (8). Dornase alfa (recombinant human DNase I) is currently used in patients with Cystic Fibrosis and reduces mucus viscosity by degrading extracellular DNA in the airways (9, 10). We proposed a trial using inhaled dornase alfa as a therapeutic target to reduce extracellular DNA and NETs in patients with acute respiratory distress syndrome (ARDS) secondary to COVID-19 pneumonia, with outcome aims including improved lung compliance and gas exchange (11).

## Methods

### Study Design

A single center, non-randomized, controlled before-and-after clinical study was designed to examine the effects of inhaled dornase alfa in patients with ARDS secondary to COVID-19 pneumonia. The Institutional Review Board at the University of Missouri approved the clinical trial including collection of clinical data and clinical samples from participating patients (trial #2022206) and case control clinical data from patients with COVID-19 infection with exemption of informed consent from each patient due to the case control portion of the study involving only information collection and analysis (#2025101). A non-randomized model was used to determine if an effect could be noticed to justify a larger randomized controlled study. Patients were recruited from the medical intensive care unit at the University of Missouri, a 250-bed academic tertiary care medical center. Recruitment was considered after confirmation of SARS-CoV-2 infection induced ARDS and progression of care requiring mechanical ventilation. Inclusion criteria included age ≥ 18 years, hospitalized, and mechanically ventilated for illness related to SARS-CoV-2 infection, with individual or surrogate ability to sign informed consent, and negative urine-based pregnancy test in female patients. Exclusion criteria included contraindication or intolerance to dornase alfa, length of mechanical ventilation expected to be less than 48 hours, life expectancy less than 24 hours based upon judgment of treatment physician, pregnancy, or inability to obtain informed consent. Patients in the treatment group received nebulized dornase alfa (2.5mL) *via* vibrating mesh through the ventilator circuit twice daily for three days after enrollment. Control group patients received standard of care for severe COVID-19 pneumonia and ARDS. No placebo group was included in this pilot study. The primary outcome measure was change in arterial oxygen saturation to inhaled fraction of oxygen (PaO_2_/FiO_2_) compared to the day of enrollment. Secondary outcomes included change in static lung compliance $\left(\frac{Tidal\;Volume\;(mL)}{Plateau\;Pressure-PEEP}\right)$ compared to the day of enrollment, duration of mechanical ventilation, length of ICU stay, length of hospitalization, secondary bacterial infections, and mortality. PaO_2_/FiO_2_ and static lung compliance measurements were all obtained when patients were supine for at least 2 hours prior to measurement. Sample size was determined based upon ability to calculate significance of the primary outcome. Interim analysis was performed every 3 patients to evaluate for trends in outcomes and consideration of trial continuation.

### Data Collection

Demographic and clinical data of the patients was obtained from electronic medical records at enrollment. Clinical study information collected included age, sex, co-morbidities, therapies received, serological testing, ventilator data, bacterial and viral culture data, days of hospitalization, days in the intensive care unit, days of mechanical ventilation, and mortality.

### Sample Acquisition and Processing

An optional part of the trial consisted of collecting patient samples including blood and bronchoalveolar lavage fluid (BALF) before and after receiving therapy with dornase alfa to compare frequency of neutrophils and NET MPO-DNA complex concentrations. BALF was either obtained from any unused fluid collected as part of routine clinical care, or if approved by the patient, additional research samples were obtained. BALF cell count and differential were all performed by the clinical laboratory with frequency of neutrophils being expressed by percent of total white blood cells per mcL and absolute cell counts per mcL. BALF used for detection of NET MPO-DNA complex concentrations were processed as follows. Mucus in BALF samples was manually broken up by gentle pipetting, followed by straining through a cell strainer, then split into 1 mL aliquots and immediately frozen at -80°C. Blood to be discarded after clinical laboratory analysis was also obtained. Approximately 1 mL blood was centrifuged at 150 g for 10 minutes at room temperature, followed by collection and freezing of the plasma at -80°C until further use. Meanwhile, neutrophils from 1 mL blood were isolated using the EasySep Human Neutrophil Enrichment Kit (Stemcell Technologies) per manufacturer instructions and diluted to 1x10^6^ cells/mL in RPMI +5% FBS. Isolated neutrophils from the blood were then seeded at 5x10^5^ cells/well of a 12 well plate (Corning), allowed to adhere for 30 minutes, followed by NET induction *via* stimulation with 10 µg/mL phorbol 12-myristate 13-acetate (PMA, Sigma) for 4 hours at 37°C. NETs were then detached from adherent cells by gentle pipetting, centrifuged at 150 g for 20 minutes at room temperature to separate NETs from cells and debris, and the supernatant was immediately frozen at -80°C. Control NETs from a healthy volunteer were prepared and stored in this same manner.

### NET MPO-DNA Complex Detection

To detect the presence of NET MPO-DNA complexes, 96-well plates (Corning) were coated with 4 µg/mL of anti-MPO monoclonal antibody (clone 4A4, Bio-Rad) in a sodium carbonate/bicarbonate buffer (pH 9.6), incubated at 4°C overnight, washed with PBS, and subsequently blocked with 1% bovine serum albumin in PBS for 30 minutes at room temperature. Thawed BALF, serum, and PMA-neutrophil-induced NET samples were immediately added after washing, followed by incubation for 90 minutes at room temperature. After washing, a 1:100 dilution of anti-DNA-POD antibody (clone MCA-33, Roche) was added to each well and incubated again for 90 minutes at room temperature. The amount of single or double-stranded DNA bound to MPO was then detected by adding 2,2’-azino-di-[3-ethylbenzthiazoline sulfonate], or ABTS substrate (Roche) and incubated for 15 minutes at room temperature on a plate shaker at 250 rpm. Plates were then read on an EPOCH microplate reader (Bio-Tek) by measuring optical density at 405 nm wavelength. MPO-DNA complexes were quantified by comparing against a standard curve of known DNA concentrations (in µg/mL) as determined by spectrophotometry from healthy volunteer control NETs prepared as described above and reported as micrograms/milliliter.

### Statistical Analysis

Statistical test and graphs were made using GraphPad Prism software. Data are presented as difference between means 95% confidence intervals. Statistical difference was assessed by two-tailed, paired or unpaired, t-test. Statistical significance for the study was defined as P-value ≤ 0.05.

### Patient Demographics

Baseline demographics and clinical characteristics of the treatment group and case control group are depicted in **Table 1**. There was no significant difference noted between the two groups regarding co-morbidities and common therapies received for COVID-19.

**Table 1:** Demographics and concurrent medical therapies of patients in the case-control group (N=20) and Dornase alfa treatment group (N=10).

Demographics	Case-Control (N=20)	Dornase alfa (N=10)	P-Value
Age (years)*	60 (24-84)	63 (47-79)	0.65
Gender**			
Male	13 (65%)	6 (60%)	0.8
Female	7 (35%)	4 (40%)	0.8
Ethnicity**			
White, non-hispanic	16 (80%)	8 (80%)	>0.999
African American	1 (5%)	0 (0%)	0.49
Hispanic/Latino	3 (15%)	2 (20%)	0.74
Co-Morbidities**			
Diabetes	11 (55%)	7 (70%)	0.45
Hypertension	14 (70%)	9 (90%)	0.24
Coronary artery	7 (35%)	5 (50%)	0.45
Disease			
Chronic lung disease	7 (35%)	3 (30%)	0.79
(COPD, asthma, ILD)			
Obesity	17 (85%)	8 (80%)	0.79
Therapies Received**			
Remdesivir	20 (100%)	9 (90%)	0.16
Corticosteroids	20 (100%)	9 (90%)	0.16
Antibiotics	19 (95%)	10 (100%)	0.49
Convalescent Plasma	11 (55%)	9 (90%)	0.06
Anticoagulation	10 (50%)	4 (40%)	0.62
Paralytics	19 (95%)	8 (80%)	0.21
Prone positioning	16 (80%)	8 (80%)	>0.999

*(mean, min to max) **(total and % of patient population). COPD, Chronic Obstructive Pulmonary Disease; ILD, Interstitial Lung Disease.

## Results

**Figure 1:**
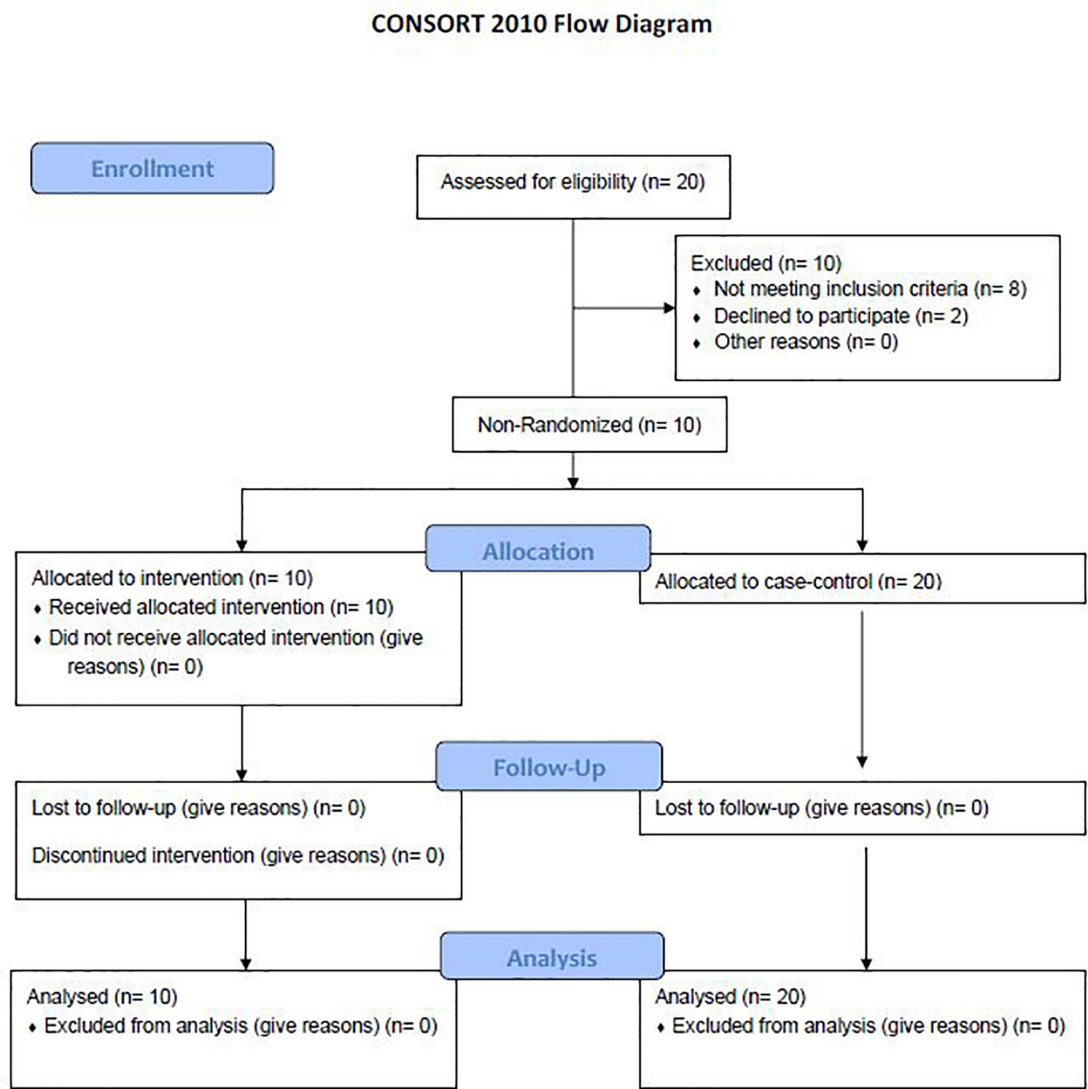
Consort flow diagram. A total of 20 patients were evaluated for the study with 8 patients not meeting inclusion criteria and 2 declining to participate. 10 patients were included in the treatment arm with no patients lost to follow up. 20 patients were randomly selected to be a part of the case-control arm (12).

**Figure 2:**
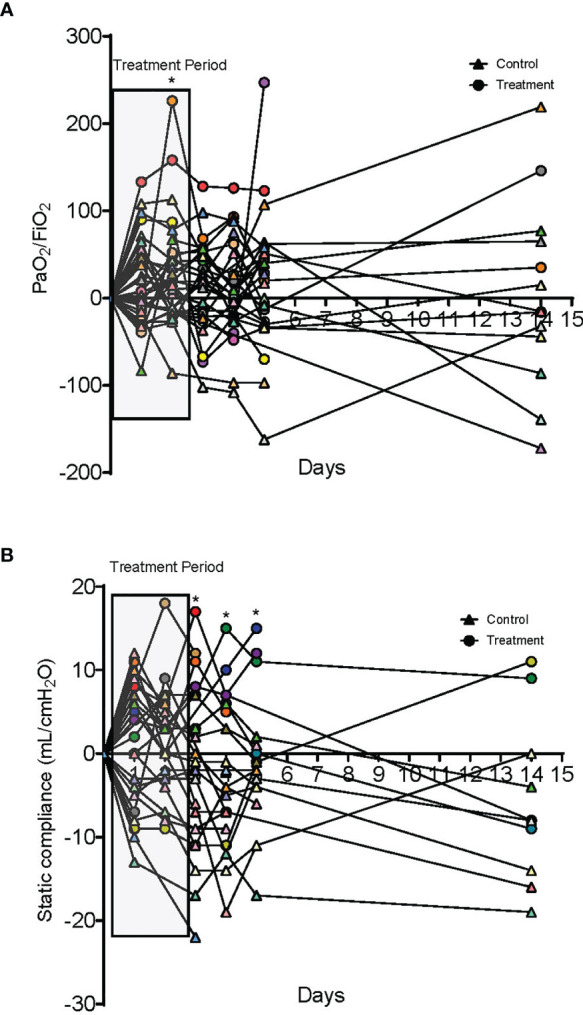
Oxygenation and compliance responses to dornase alfa treatment. **(A)** Change in PaO_2_/FiO_2_ compared to day 0. Each colored circle symbol represents a unique patient in the treatment group and each colored triangle symbol represents a unique patient in the control group. A significant increase is noted in the treatment group at day 2 of treatment with a non-statistically significant increase for the additional 14 days. **(B)** Change in static lung compliance (mL/cmH_2_O) compared to day 0. Each colored circle symbol represents a unique patient in the treatment group and each colored triangle symbol represents a unique patient in the control group. Starting on day 3 there was a significant improvement in lung compliance in the treatment group compared to the case control group that was sustained to day 5. No statistically significant difference was noted at 14 days.

**Figure 3:**
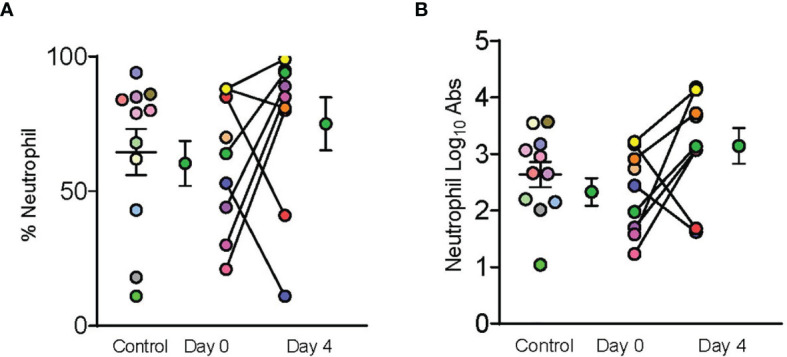
Dornase alfa treatment did not reduce BALF neutrophils. Control, day 0 and day 4 symbols are displayed as mean ± SEM of 11 subjects in the control group and 9 subjects in the treatment group. Each individual color-coded point with connecting line between day 0 and 4 represents a unique patient in the treatment group (N=9). Two patients only had samples from a single time point due to sample degradation. Only 11 subjects in the control group underwent BALF sampling during hospitalization and are represented by individual colored coded data points. No statistically significant difference was noted in **(A)** % Neutrophils, or **(B)** log transformed absolute neutrophil counts in the bronchoalveolar fluid between case control patients and patients prior to receiving dornase alfa.

**Figure 4:**
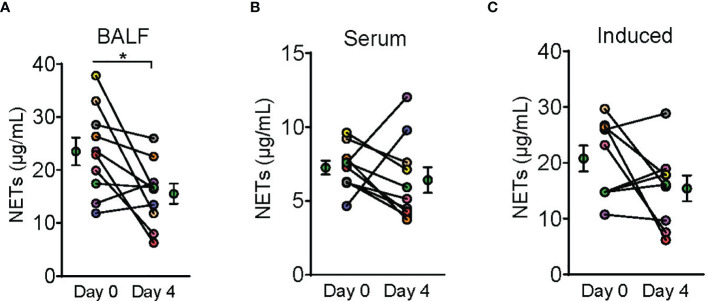
Dornase alfa treatment reduced BALF NETs. Symbols on Day 0 and Day 4 are represented as mean ± SEM of 10 subjects in the bronchoalveolar fluid (BALF) analysis and serum neutrophil extracellular traps (NETs) analysis and 9 subjects in the induced NETs analysis. Due to not having the appropriate material prior to sample collection from the first enrolled patient, we were only able to perform induced NET analysis on 9 subjects **(C)**. Each connecting line with points at Day 0 and 4 represents a unique patient sample and is patient colored coded to previous graphs (N=10). NETs defined by NET MPO : DNA complex measurement was significantly reduced in the BALF after 3 days of therapy with dornase alfa **(A)**. There was no significant change in serum NETs or NETs induced *in vitro* from patient neutrophils harvested after dornase alfa therapy **(B, C)** respectively.

A total of 10 dornase alfa-treated patients were included in the pilot trial and all 10 were included in the final analysis. As the trial was not established as a randomized trial, a total of 20 case control patients were also included in the final analysis (**Figure 1**). Recruitment for the study was performed from June 19, 2020 to December 1, 2020. A significant improvement in change of PaO_2_/FiO_2_ from baseline was noted in the treatment group compared to change of PaO_2_/FiO_2_ from baseline in case-control subjects at day 2 (**Figure 2A**; Difference between means 95% CI, 2.96 to 95.66, P-value = 0.038). The significant difference in measured PaO2/FiO2 was not maintained after drug treatment completion with measurements obtained out to 14 days on mechanical ventilation. Improvement was also noted in static lung compliance at days 3 through 5, which again was not sustained durably beyond the drug treatment period out to 14 days (**Figure 2B**; Difference between means 95% CI, 4.8 to 19.1, 2.7 to 16.5, and 5.3 to 19.2 mL/cmH2O respectively). Secondary outcomes including adverse events did not show statistically significant differences between treatment and case-control groups assessed up to 90 days (**Table 2**). BALF and blood samples obtained in the treatment group before and after therapy were analyzed for cell differential and accumulation of MPO-DNA complexes. Eleven patients in the control group also underwent diagnostic bronchoscopy and samples in the treatment group were also compared to this control group. There was no difference in neutrophil counts or percentages between the before treatment samples and the control group (**Figure 3**). Despite the non-significant increase in neutrophil percentage and absolute log_10_ neutrophil count (**Figure 3**), there was a significant reduction in BALF NETs measured as MPO-DNA complexes (Difference between means 95% CI, -14.7 to -1.32, P-value = 0.01) (**Figure 4A**) after therapy with inhaled dornase alfa. There was no difference in the serum MPO-DNA activity before or after therapy, nor in NETs that were experimentally induced from neutrophils isolated from patient blood that were treated with PMA *ex vivo* (**Figures 4B, C**).

**Table 2:** Primary and Secondary Outcomes of patients in case-control group (N=20) and Dornase alfa treatment group (N=10).

Primary and Secondary Outcomes			
	Control (N = 20)	Dornase alfa (N = 10)	P-Value
**Primary Outcomes**			
ΔPaO_2_/FiO_2_ from Day 0 (95% CI)			
Day 1	-0.8 to 43.6	-29.7 to 51.1	0.59
Day 2	-9.6 to 33.1	5.2 to 117	**0.04**
Day 3	-9.7 to 34.7	-21 to 68	0.59
Day 4	-24.8 to 31.7	-21.8 to 70.1	0.38
Day 5	-28.8 to 38.8	-35.9 to 111	0.32
Day 14	-92.6 to 70.2	-150.8 to 260.8	0.38
**Secondary Outcomes**			
Δ Lung compliance from Day 0 (95% CI)			
Day 1 (mL/cmH_2_O)	-4.2 to 3.9	-1.7 to 7.9	0.28
Day 2	-3.1 to 2.3	-1 to 9	0.08
Day 3	-9.8 to -1.6	-0.8 to 13.3	**<0.01**
Day 4	-9.4 to -1.8	-3.2 to 11.3	**<0.01**
Day 5	-8.4 to -1.3	-1.8 to 16.6	**<0.01**
Day 14	-17.9 to -2.4	-16.3 to 17.8	0.09
Δ PEEP from Day 0 (95% CI)			
Day 1 (cmH_2_O)	-0.7 to 1.7	-0.8 to 1.2	0.74
Day 2	-0.8 to 1.7	-1.7 to 0.9	0.34
Day 3	-1.1 to 2.4	-3.1 to 1.3	0.27
Day 4	-1.6 to 2.2	-4.5 to 1.6	0.28
Day 5	-1.4 to 3.1	-5.5 to 2	0.19
Days on Mechanical Ventilation*	18.2 (8-38)	15.2 (5-29)	0.47
Days in ICU*	22.1 (11-47)	16.5 (7-30)	0.23
Days of Hospitalization*	28.7 (15-60)	22.5 (8-52)	0.61
Secondary Pulmonary Infections**	5 (25%)	3 (30%)	0.78
Mortality, 28-day**	9 (45%)	4 (40%)	0.8
Mortality, 90-day**	11 (55%)	4 (40%)	0.46

*(mean, min to max) **(total and % of patient population). ICU, Intensive Care Unit. Significant P-values are in bold.

## Discussion

Neutrophils play an important role in the innate immune response in the lungs and have been shown to be higher in BALF in patients with COVID-19, particularly those with increased disease severity (11, 13). An associated increase in neutrophil chemoattractants have also been observed (8). Neutrophils can release complexes of chromosomal DNA, histones and granule enzymes, such as MPO, as NETs trapping microorganisms, but also causing collateral damage in the form of thrombosis and lung injury (13, 14). Earlier studies noted an increase in circulating markers for NETs in patients with COVID-19 compared to healthy controls, as well as autopsy findings have confirmed the presence of NETs in the lungs (5, 15). The concept of NET-driven lung injury is not unique to COVID-19. Targeting NETs in ARDS has been applied to other causes of pneumonia as the level of NETs has been associated with disease severity (16, 17).

Our group began by applying dornase alfa to one severe COVID-19 patient who subsequently showed improvement in oxygenation after treatment (2). Therefore, we designed this initial clinical trial with PaO_2_/FiO_2_ improvement as the primary outcome objective. We predicted that the inhaled drug would decrease extracellular DNA and NETs in the lung and improve oxygenation. Currently, the data appear to support both predictions. There was a decrease in MPO-DNA complexes, which suggests a decrease in NETs (**Figures 2**–**4**). This result makes sense given the accumulation of neutrophils in the lungs in response to infection, with lytic death and DNA release having deleterious effects. At the same time, the ability of dornase alfa to degrade DNA and improve oxygenation wanes rapidly upon drug withdrawal. A more durable effects of decreased magnitude may be possible to determine upon increasing patient N in a more extensive trial. One concern was the potential negative effect of degrading NETs whose function is in part to fight off invading pathogens. There was no observed significant increase in secondary pulmonary infections. We conclude that the premise of applying dornase alfa to degrade DNA in NETs and improve oxygenation in severe COVID-19 induced ARDS appears sound and should be further explored in a more extensive clinical trial. We also propose that the time of drug administration be extended, predicting that a greater duration of beneficial effects may thereby be induced, with the goal of decreasing morbidity and mortality rates in these patients. There is currently a phase 2 open-label, randomized, Best-Available-Care (BAC) and historic-controlled trial ongoing at University College London Hospital looking into the effects of inhaled dornase alfa over 7 days on the outcome of patients admitted to the hospital who are at risk of ventilatory failure (18).

## Limitations

Our study is limited by being a single center study and a small sample size which does not allow for power to determine many of the secondary outcomes. If plausible, future studies should aim to include multi-center patient data sets and a larger patient population. Our population was also predominantly white, non-Hispanic and may underrepresent the effect on other ethnic groups. Standard of care for severe COVID-19 patients includes treatment with systemic corticosteroids, which has been reported to reduce systemic NET activity (16). The limited serological change in MPO-DNA activity may have been affected by this therapy. Another limitation is that MPO-DNA complexes in patient samples were discussed as known NET products, but the mechanism of their generation was not determined.

## Conclusion

Severe COVID-19 pneumonia leading to ARDS is associated with increased extracellular MPO-DNA complexes, potentially as NETs, in the alveolar space. Inhaled Dornase alfa, *via* degradation of alveolar DNA, significantly improved oxygenation and lung compliance. Due to a persistent inflammatory state of the lungs in COVID-19 and ARDS, the effect is short lived after drug removal. Results of our pilot study warrant further consideration of a larger randomized trial in patients with ARDS secondary to COVID-19, as well as other causes of ARDS.

## Data Availability Statement

The raw data supporting the conclusions of this article will be made available by the authors, without undue reservation.

## Ethics Statement

The studies involving human participants were reviewed and approved by University of Missouri Institutional Review Board. The patients/participants provided their written informed consent to participate in this study.

## Author Contributions

ZH, AK, AS, AE, and MA collected and analyzed the data. ZH, AE, and AS wrote the manuscript. AS, AE, AK, L-AA, and MA reviewed/edited the manuscript. All authors contributed to the article and approved the submitted version.

## Funding

Funding was provided by the University of Missouri Division of Pulmonary and Critical Care Research Fund.

## Conflict of Interest

The authors declare that the research was conducted in the absence of any commercial or financial relationships that could be construed as a potential conflict of interest.

## Publisher’s Note

All claims expressed in this article are solely those of the authors and do not necessarily represent those of their affiliated organizations, or those of the publisher, the editors and the reviewers. Any product that may be evaluated in this article, or claim that may be made by its manufacturer, is not guaranteed or endorsed by the publisher.

## References

[1] Emanuel EJ Persad G Upshur R Thome B Parker M Glickman A. Fair Allocation of Scarce Medical Resources in the Time of Covid-19. N Engl J Med (2020) 382(21):2049–55. doi: 10.1056/NEJMsb2005114 32202722

[2] Earhart AP Holliday ZM Hofmann HV Schrum AG. Consideration of Dornase Alfa for the Treatment of Severe COVID-19 Acute Respiratory Distress Syndrome. New Microbes New Infect (2020) 35:100689. doi: 10.1016/j.nmni.2020.100689 32355564 PMC7192073

[3] Brinkmann V Reichard U Goosmann C Fauler B Uhlemann Y Weiss DS. Neutrophil Extracellular Traps Kill Bacteria. Science (2004) 303(5663):1532–5. doi: 10.1126/science.1092385 15001782

[4] DeLeo FR Allen LH. Phagocytosis and Neutrophil Extracellular Traps. Fac Rev (2020) 9:25. doi: 10.12703/r/9-25 33659957 PMC7886055

[5] Barnes BJ Adrover JM Baxter-Stoltzfus A Borczuk A Cools-Lartigue J Crawford JM. Targeting Potential Drivers of COVID-19: Neutrophil Extracellular Traps. J Exp Med (2020) 217(6):e20200652. doi: 10.1084/jem.20200652 32302401 PMC7161085

[6] Schönrich G Raftery MJ. Neutrophil Extracellular Traps Go Viral. Front Immunol (2016) 7:366. doi: 10.3389/fimmu.2016.00366 27698656 PMC5027205

[7] Kambas K Chrysanthopoulou A Vassilopoulos D Apostolidou E Skendros P Girod A. Tissue Factor Expression in Neutrophil Extracellular Traps and Neutrophil Derived Microparticles in Antineutrophil Cytoplasmic Antibody Associated Vasculitis may Promote Thromboinflammation and the Thrombophilic State Associated With the Disease. Ann Rheum Dis (2014) 73(10):1854–63. doi: 10.1136/annrheumdis-2013-203430 23873874

[8] Middleton EA He XY Denorme F Campbell RA Ng D Salvatore SP. Neutrophil Extracellular Traps Contribute to Immunothrombosis in COVID-19 Acute Respiratory Distress Syndrome. Blood (2020) 136(10):1169–79. doi: 10.1182/blood.2020007008 PMC7472714 32597954

[9] Shak S Capon DJ Hellmiss R Marsters SA Baker CL. Recombinant Human DNase I Reduces the Viscosity of Cystic Fibrosis Sputum. Proc Natl Acad Sci USA (1990) 87(23):9188–92. doi: 10.1073/pnas.87.23.9188 PMC55129 2251263

[10] Ramsey BW Astley SJ Aitken ML Burke W Colin AA Dorkin HL. Efficacy and Safety of Short-Term Administration of Aerosolized Recombinant Human Deoxyribonuclease in Patients With Cystic Fibrosis. Am Rev Respir Dis (1993) 148(1):145–51. doi: 10.1164/ajrccm/148.1.145 8317790

[11] Wang Q Doerschuk CM Mizgerd JP. Neutrophils in Innate Immunity. Semin Respir Crit Care Med (2004) 25:33–41. doi: 10.1055/s-2004-822303 16088447

[12] Schulz KF Altman DG Moher D CONSORT Group. CONSORT 2010 Statement: Updated Guidelines for Reporting Parallel Group Randomised Trials. BMJ (2010) 340:c332. doi: 10.1136/bmj.c332 PMC2844940 20332509

[13] Zhou Z Ren L Zhang L Zhong J Xiao Y Jia Z. Heightened Innate Immune Responses in the Respiratory Tract of COVID-19 Patients. Cell Host Microbe (2020) 27(6):883–90.e2. doi: 10.1016/j.chom.2020.04.017 32407669 PMC7196896

[14] Frantzeskaki F Armaganidis A Orfanos SE. Immunothrombosis in Acute Respiratory Distress Syndrome: Cross Talks Between Inflammation and Coagulation. Respiration (2017) 93(3):212–25. doi: 10.1159/000453002 27997925

[15] Zuo Y Yalavarthi S Shi H Gockman K Zuo M Madison JA. Neutrophil Extracellular Traps in COVID-19. JCI Insight (2020) 5(11):e138999. doi: 10.1172/jci.insight.138999 PMC7308057 32329756

[16] Ebrahimi F Giaglis S Hahn S Blum CA Baumgartner C Kutz A. Markers of Neutrophil Extracellular Traps Predict Adverse Outcome in Community-Acquired Pneumonia: Secondary Analysis of a Randomised Controlled Trial. Eur Respir J (2018) 51:1701389. doi: 10.1183/13993003.01389-201 29519921

[17] Bendib I de Chaisemartin L Granger V Schlemmer F Maitre B Hüe S. Neutrophil Extracellular Traps Are Elevated in Patients With Pneumonia-Related Acute Respiratory Distress Syndrome. Anesthesiology (2019) 130(4):581–91. doi: 10.1097/ALN.0000000000002619 30676417

[18] Nebulised Dornase Alfa for Treatment of COVID-19 (COVASE). ClinicalTrials.gov Identifier NCT04359654. Updated October 19 (2020). Available at: https://clinicaltrials.gov/ct2/show/NCT04359654?term=NCT04359654&draw=2&rank=1 (Accessed August 24, 2021).

